# Reply to: “Critique on conclusions regarding toxic compounds in *Jatropha curcas* kernel cake”

**DOI:** 10.1038/s42003-021-02871-y

**Published:** 2021-12-01

**Authors:** Changhe Zhang, Xing-Hong Wang

**Affiliations:** 1 Center for the Research and Technology of Agro-Environmental and Biological Sciences (CITAB)/Department of Biology and Environment, Universidade de Trás-os-Montes e Alto Douro (UTAD), Apartado 1013, 5001-801 Vila Real, Portugal; 2 Instituto de Tecnologia Química e Biológica António Xavier (Green-it Unit), Universidade Nova de Lisboa, 2780-157 Oeiras, Portugal; 3 Yunnan Institute of Microbiology, School of Life Science, Yunnan University, Kunming, 650091 Yunnan P. R. China

**Keywords:** Biological techniques, Biotechnology, Pathogenesis

**replying to** G. Francis et al. *Communications Biology* 10.1038/s42003-021-02869-6 (2021)

We are honored to have received a critique raised by Dr. George Francis et al. (hereinafter referred to as peers or peer) on the conclusion of our paper^1^. We highly appreciate the critique and welcome all comments and critiques on our paper because we believe that constructional critique also helps promote scientific advance and technological development. However, we have to say that the current critique is not based on convincing evidence, but only opinions of the peers.

In the critique, Francis et al. did not provide any new experiment results, but cited 15 publications to defend an opinion that phorbol esters (PEs) are the major toxic compounds in Jatropha seed/kernel cake, and charged our conclusion mainly by questioning the origin of the kernel cake.

## Convincing evidence for the peers’ opinion is unavailable

Peers’ first quotation “*Phorbol esters are absent in jatropha seed cake*” is not a sentence in our papers nor our conclusion nor opinion; we never exclude the possible existence of PEs in Jatropha seed/kernel cake. However, the existence of PEs in Jatropha seeds and/or seed/kernel cake does not mean that PEs are the principal toxins of the cake. Convincing evidence for the peers’ opinion is not available. The actual content of the 15 references does not support their opinion, but rather, refutes their opinion as can be seen in the following.

First, PEs have never been shown purified or accurately determined by valid measures from Jatropha seed/kernel cake in any reports. The peers did not cite any reference to charge the fact that no PEs have ever been isolated from Jatropha seed cake in any publications. They cited References 2–4^2–4^ to “*show that phorbol esters have been detected in deoiled Jatropha cake*”. In fact, the researchers just used HPLC to “measure” PEs in Jatropha seeds and/or seed cake^2,4^, or in the seeds rather than seed cake (page 3151)^3^, respectively, in the absence of an authentic standard, which is not a valid method as shown in the following section.

Peers cited References 5^5^, 6^6^, and 7^7^ to “*show that phorbol esters are indeed present in Jatropha kernel meal and that they are the toxic components in Jatropha cake and kernel meal*.” In fact, the concentration of PEs in the cake or other materials “determined” by HPLC in the absence of an authentic standard was the only proof of the presence of PEs and of the toxicity level in these papers^5–7^; while the methanol extract of seed oil^5,6^, or, “detoxified Jatropha kernel meal”^7^, rather than any purified PEs, was directly used to do the research work concerning the toxicity of PEs. The consideration that the toxicity of Jatropha cake or the extract of Jatropha oil equals to the toxicity of PEs is too subjective to believe.

Peers cited References 8–11^8–11^ to support their opinion via the comparison of PEs between the toxic and non-toxic varieties. Actually, peer Makkar’s publications^8,9^ demonstrate that peers’ claim “*the conventional toxic variety of Jatropha seed kernels differ from the edible, non-toxic variety only in the presence of PEs”* or *“In the papers published prior to that of Wang et al.1, phorbol esters were conclusively shown to be the toxic principle of Jatropha (……), whose presence or absence makes Jatropha seed kernels non-edible or edible, respectively*.” is a false opinion rather than a finding or conclusion. In Reference 8^8^, the kernel of the non-toxic variety also contained PEs at as high as 0.11 mg/g^8^; while the content of the major antinutrients in the toxic variety was double that of the non-toxic variety (tannins—0.04% *vs* 0.02%, lectin—102 *vs* 51 mg/ml, page 213). Reference 9 only compared some “non-toxic” seeds purchased from seven farmers: some seeds contained PEs, others didn’t (page 35), but all the seeds were sold for human comsumption^9^. Reference 10 did not contain any research on PEs or antinutrients^10^. Reference 11^11^ seems favoring to their opinion, while the PEs were also determined without any authentic standard.

Concerning peers’ claims “*The individual PEs have also been separately extracted, purified, structure determined, and studied for toxicity as can be seen in previous publications13,15*”, Reference 13^12^ claimed to have obtained Jatropha C1 from Jatropha oil, but didn’t contain any toxicity studies^12^. However, because the authors did not show either any data or any spectra of the NMR^12^ the identity of the Jatropha factor C_1_ has to be questioned. Actually, Reference 15 did not contain either purification, structure determination, or toxicity studies of any PEs^13^.

## Determination of PEs using HPLC with TPA as standard is not reliable

Peers cited References 3^3^, 11^11^, 12^14^, 13–16^12,13,15,16^ to show the validity of the determination of Jatropha PEs by HPLC. It is well known that HPLC only has the possibility to identify a compound when there is an authentic standard, by verifying the retention time *t*_R_ with the standard. All the references cited by the peers mentioned above used TPA/PMA as standard to determine 4–6 Jatropha factors, except for Reference 13^12^, in which the authors claimed using TAP and Jatropha factor C_1_ obtained from oil as standard, while the identity of the Jatropha C1 was not confirmed by NMR data or spectra^12^, as mentioned previously.

The UV spectrum and *t*_R_ of TPA and Jatropha factors are quite different. TPA contains a peak with *λ*_max_ of 242 nm^11^, while Jatropha factor C_1_, C_2_, C_3_, C_4_ + C_5_, and C_6_ contain a peak of *λ*_max_ of 284, 280, 272, 290 + 303 + 317, and 276 nm, respectively^17^. How to accurately quantify the six individuals of Jatropha PEs as TPA equivalents? What is the reference for accurately identifying a peek appeared in the chromatograms at 280 or 240 nm being or not being a Jatropha factor? What is the reference for accurately discriminating the six individuals of the Jatropha factors? We cannot find any answer to these basic questions from the citations. Actually, by comparing UV detection and MS detection Neu et al. also pointed out “*a much smaller absorption at 280* *nm than the PEs resulting in wrong values when using PMA as standard*” (page 5)^16^.

The peers claimed “*the phorbol esters are detected based on a characteristic peak structure in the chromatogram*”. However, UV spectrum is not determinative in molecular structure identification. Therefore, Haas et al., did not publish the authentic UV spectra nor the peak structure of the individual Jatropha factors in the chromatogram on HPLC^17^. In fact, both the peak numbers 4^3^, 3–4^12^, 5^13^, 6^11,15^, and peak structures/shapes of the PEs in the HPLC chromatogram shown in the citations were different; different column and elution conditions gave different peak numbers and structures^12^. Therefore, the “*characteristic peak structure”* claimed by the peers did not exist in the citations.

In Reference 12 of the critique, the reviewers of EFSA also concluded that there were no valid measures available to quantify PEs (page 53) and that there is a need for standards for individual Jatropha PEs and for analytical methods validated for the quantification of Jatropha PEs (page 56)^14^.

As peer Makkar expressed, the determination of PEs by HPLC using TPA as standard is an estimation (page 316)^7^. Therefore, in the absence of authentic standards HPLC alone is not sufficient for the determination of Jatropha PEs, the combination with MS and NMR is essential. In conclusion, PEs are the major toxic components of Jatropha seed/kernel cake is lack of convincing evidence.

## No problem in the materials nor conclusion of our work

The seeds and kernel cake used in our work did not have the problem assumed by the peers. The origin of the seeds and kernel cake was presented in a previous paper of ours—the first citation of our paper^18^. The seeds of *J. curcas* were produced in Chuxiong (101°63′E, 24°70′N), Yunnan, China. Clean mature seeds were de-hulled to isolate the kernels. The oil of the stir-fried kernels was extracted by mechanical press in order to obtain kernel cake and oil. The freshly produced kernel cake (oil residue about 2%), provided by Yunnan Shenyu New Energy Company Limited (Kunming, China), was directly used in our works. The seeds belong to the toxic “Savanna-type” provenance^19^.

Our result that no PEs were isolated from the major toxic fractions of the methanol extract of as much as 2000 kg Jatropha kernel cake is in accordance with the fact that, so far, no PEs have ever been isolated from any Jatropha seed/kernel cake, no matter toxic or non-toxic. In the papers published prior to ours PEs were considered or assumed as, rather than conclusively shown, to be the toxic principle of Jatropha cake because convincing evidence on the opinion is not available, as shown in the previous two sections. Our literature surveys also revealed no report prior to ours on the occurrence of hydroxy-octadecenoic acids (HOEAs) in any plant part of *J. curcas*. However, that the peers in this area did not know the existence of HOEAs in Jatropha plants neither affects their existence nor is sufficient to deny our findings.

Our toxin isolation process from both the cake and seed oil was focused on all the major toxins, which was monitored/directed by live carp fingerling toxicity tests, not only on PEs^1^. That’s the main reason why we could isolate the HOEAs while other peers could not. The toxin extraction from seed oil, separation and molecular structure identification processes were provided in detail in our paper. The major toxic components purified from the oil were also confirmed to be the HOEAs rather than PEs^1^. By the toxicity-directed toxin separation process, in addition to HOEAs, we also purified some other toxins from the kernel cake, except for PEs^1,20^. Therefore, it is not reasonable for us to include the analysis of PEs in oil in our paper as the peers requested.

The peers presented the references in the critique in a form that favors to their opinions, while the actual content of the references did not support or rather refuted their opinions, as shown previously. Our conclusion was based on our systemic findings that established a solid chain of evidence: the major toxic components purified from the kernel cake extract with methanol were identified as HOEAs rather than PEs; HOEAs caused a similar toxicity on animals to that caused by Jatropha seeds and/or seed/kernel cake; the molecular basis and underlying mechanism of the toxicity of HOEAs were deciphered^1^.

## References

[1] Wang, X. H. et al. Hydroxy-octadecenoic acids instead of phorbol esters are responsible for the Jatropha curcas kernel cake’s toxicity. *Commun. Biol*. **3**, 228 (2020). 10.1038/s42003-020-0919-z PMC7210109 32385384

[2] Faria-Machado, A. F. et al. Method validation for analysis of phorbol esters from *Jatropha curcas*. *Ind. Crop. Prod.* **140**, 111627 (2019).

[3] Makkar HPS, Becker K, Sporer F, Wink M (1997). Studies on nutritive potential and toxic constituents of different provenances of Jatropha curcas. J. Agr. Food Chem..

[4] Li Y (2018). Substitution of soybean meal with detoxified *Jatropha curcas* kernel meal: effects on performance, nutrient utilization, and meat edibility of growing pigs. Asian-Australas. J. Anim. Sci..

[5] Becker K, Makkar HPS (1998). Effects of phorbol esters in carp (Cyprinus carpio L). Vet. Hum. Toxicol..

[6] Devappa RK, Makkar HPS, Becker K (2010). Biodegradation of Jatropha curcas phorbol esters in soil. J. Sci. Food Agr..

[7] Kumar V, Makkar HPS, Becker K (2011). Detoxified Jatropha curcas kernel meal as a dietary protein source: growth performance, nutrient utilization and digestive enzymes in common carp (Cyprinus carpio L.) fingerlings. Aquacult Nutr..

[8] Makkar HPS, Aderibigbe AO, Becker K (1998). Comparative evaluation of non-toxic and toxic varieties of Jatropha curcas for chemical composition, digestibility, protein degradability and toxic factors. Food Chem..

[9] Makkar HPS, Becker K, Schmook B (1998). Edible provenances of *Jatropha curcas* from Quintana Roo state of Mexico and effect of roasting on antinutrient and toxic factors in seeds. Plant Food Hum. Nutr..

[10] Valdes-Rodriguez OA, Sanchez OS, Perez-Vazquez A, Caplan J (2013). The Mexican non-toxic *Jatropha curcas* L., food resource or biofuel?. Ethnobot. Res Appl.

[11] He W (2011). Analysis of seed phorbol-ester and curcin content together with genetic diversity in multiple provenances of *Jatropha curcas* L. from Madagascar and Mexico. Plant Physiol. Bioch.

[12] Devappa RK, Bingham JP, Khanal SK (2013). High performance liquid chromatography method for rapid quantification of phorbol esters in Jatropha curcas seed. Ind. Crop Prod..

[13] Baldini M (2014). Determination of phorbol esters in seeds and leaves of Jatropha curcas and in animal tissue by high-performance liquid chromatography tandem mass spectrometry. Ind. Crop Prod..

[14] Panel EC (2015). Opinion: Risks for human and animal health related to the presence of phorbol esters in Jatropha kernel meal. EFSA J..

[15] Roach JS, Devappa RK, Makkar HPS, Becker K (2012). Isolation, stability and bioactivity of Jatropha curcas phorbol esters. Fitoterapia.

[16] Neu, P. M., Schober, S. & Mittelbach, M. Quantification of phorbol esters in *Jatropha curcas* by HPLC-UV and HPLC-ToF-MS with standard addition method. *Eur. J. Lipid Sci. Tech.* **120**, 1800146 (2018).

[17] Haas W, Sterk H, Mittelbach M (2002). Novel 12-deoxy-16-hydroxyphorbol diesters isolated from the seed oil of *Jatropha curcas*. J. Nat. Prod..

[18] Wang XH (2013). Detoxification of *Jatropha curcas* kernel cake by a novel *Streptomyces fimicarius* strain. J. Hazard Mater..

[19] Yang CY, Fang G, Long YF (2012). Review and prospects of Jatropha biodiesel industry in China. Renew. Sust. Energ. Rev..

[20] Wang XH, Liu L, Li J, Wu SH (2018). Chemical constituents of the seed cake of *Jatropha curcas*. Chem. Nat. Compd..

